# Escalating Risks: Injury Patterns in Escalator-Related Trauma

**DOI:** 10.7759/cureus.74535

**Published:** 2024-11-26

**Authors:** Jacob L Guimond, Austin Henken-Siefken, Andrew McCague

**Affiliations:** 1 General Surgery, Desert Regional Medical Center, Palm Springs, USA; 2 Surgery, Desert Regional Medical Center, Palm Springs, USA; 3 Trauma and Acute Care Surgery, Desert Regional Medical Center, Palm Springs, USA

**Keywords:** elderly falls, fall from height, fall injury, fall-related trauma, trauma center

## Abstract

Falls from escalators, although infrequent, can result in a wide array of injuries, ranging from minor lacerations to fatal outcomes. This retrospective study aims to detail the treatment and outcomes of eight patients who sustained blunt-force trauma from escalator falls and received care at Desert Regional Medical Center in Palm Springs, California. Among these eight patients, seven required hospitalization, with three necessitating intensive care unit (ICU) admission, and two patients ultimately succumbing to their injuries. This investigation provides a comprehensive overview of the injury patterns associated with escalator falls, the medical interventions implemented, and the resultant patient outcomes. The findings aim to inform strategies for improving preventative measures and optimizing patient care.

## Introduction

Falls are the leading cause of unintentional injury in individuals aged 65 and older [1]. In 2020, nearly one in four adults over the age of 65 reported experiencing a fall, which highlights the significant prevalence of this issue [2]. Furthermore, the rate of unintentional falls was over five times higher in adults aged 75 and older when compared to adults between the ages of 65 to 74 years [3]. It has also been found that falls are the most common mechanism of injury leading to traumatic brain injury (TBI) across all ages, with the second highest incidence of TBI across all ages occurring in people aged 85 years and older [4]. The prevalence of escalator-related injuries has become a growing concern in public health, particularly in urban environments as buildings increasingly rely on escalators for pedestrian transportation. Escalators pose a greater risk for the elderly population especially, given their increased risk of falls. It has also been found that elderly individuals are more likely to fall from an escalator, with one study showing that 53% of cases were in individuals greater than 60 years old [5]. In Palm Springs, the median age for residents is 57.3 years old and 33% of residents are older than 64 years old [6]. These figures are nearly two times the average for California, where the median age is 37.3 years and only 15% of Californian residents are over the age of 64. These demographics, along with the presence of an airport and eight casinos within an hour drive of Palm Springs, all of which utilize escalators, pose a potentially greater risk of traumatic injury to the population.

Elderly patients have also been shown to experience more severe outcomes from falls due to age-related factors. One factor is a decline in bone mineral density, which increases the likelihood of sustaining a fracture from a fall. Another factor leading to more severe outcomes is frailty, characterized by increased vulnerability to stressors due to decreased muscle strength and physiologic reserve, leading to decreased energy available to respond to stressors. Furthermore, comorbidities can also play a significant role in the rate of falls in the elderly. Conditions such as arthritis, visual impairment, and cognitive decline can also increase a person’s risk of falling. In the United States, it has been shown that 30% of people over the age of 65 will experience a fall each year [7]. Consequences of these falls often result in fractures, head injuries, and a decline in functional independence. Falls have also been associated with increased risk of mortality in elderly individuals, with one study showing that patients aged 70-74 had a significantly greater mortality rate than any younger age group [8]. Another study showed that people 65 years and older had a two-fold increase in mortality risk after experiencing a traumatic injury, and also spent significantly longer times hospitalized than younger age groups [9].

The location and setting of escalators also pose an increased risk of injury. Most patients who presented to the Desert Regional Medical Center (DRMC) Emergency Department sustained their injuries at one of the many casinos located in the Coachella Valley. This setting may be associated with increased alcohol use, leading to impaired balance and coordination. Studies have shown that there is a positive correlation between falls and alcohol intoxication [10]. If the proper safety measures are not in place, this could lead to significant harm.

The aim of this retrospective study is to present eight cases of individuals who experienced falls from an escalator and received care at DRMC. We will outline the injury patterns, length of hospital stay, length of intensive care unit stay if applicable, and disposition on discharge.

## Materials and methods

We conducted a retrospective cohort study at DRMC in Palm Springs, California. Approval was obtained from MetroWest Medical Center Institutional Review Board (IRB approval number: 2024-023) prior to the initiation of this study. This study adhered to ethical standards and ensured the confidentiality of patient information throughout the research process.

The DRMC trauma registry includes a total of 3,145 patients consolidated into an Excel spreadsheet (Microsoft, Redmond, WA, USA). The DRMC trauma registry was queried to identify patients who had received care at this facility after sustaining injuries related to falls from an escalator. The search criteria included all patients treated for escalator-related injuries over a specified time period, which spanned from January 2017 to December 2022. From this dataset, eight patients were identified who met the criteria for inclusion in our study. No exclusion criteria were necessary for patient selection, allowing for a complete representation of all patients who met the inclusion criteria. The data utilized in this study is routinely collected by DRMC and includes a wide range of variables, including patient demographics, past medical history, injury patterns, and disposition upon discharge. A comprehensive chart review was performed to gather detailed information regarding each patient’s injury patterns upon presentation to the emergency department, interventions taken for care, hospitalization course, and treatment outcomes at our Level 1 trauma center. This analysis aimed to identify trends and correlations within the data, contributing to a better understanding of the impact of falls from escalators on older adults.

## Results

​​​​​​Data gathered between 2017 to 2022 showed eight patients (six female, two male) ranging from 70 to 96 years old who presented with injuries related to falling from an escalator. Their hospital course and outcomes are detailed further here.

Patient 1 is a 96-year-old female with no known past medical history who presented to the DRMC after falling backward on an escalator, resulting in multiple skin avulsions to the upper and lower extremities. Upon admission, patient was unable to answer questions due to pain, however emergency medical services denied any loss of consciousness. X-ray and CT imaging taken during the hospital stay showed no signs of fracture or acute hemorrhage. Surgical debridement of the wounds was completed with negative pressure therapy placed, and plastic surgery was consulted for repair of the lacerations. She was admitted to the intensive care unit by trauma surgery services, where she stayed for three days before being transferred to medical/surgical floor for four more days. After seven total days in the hospital, she was discharged to a skilled nursing facility for rehabilitation, with expectations to return to previous level of function.

**Figure 1:**
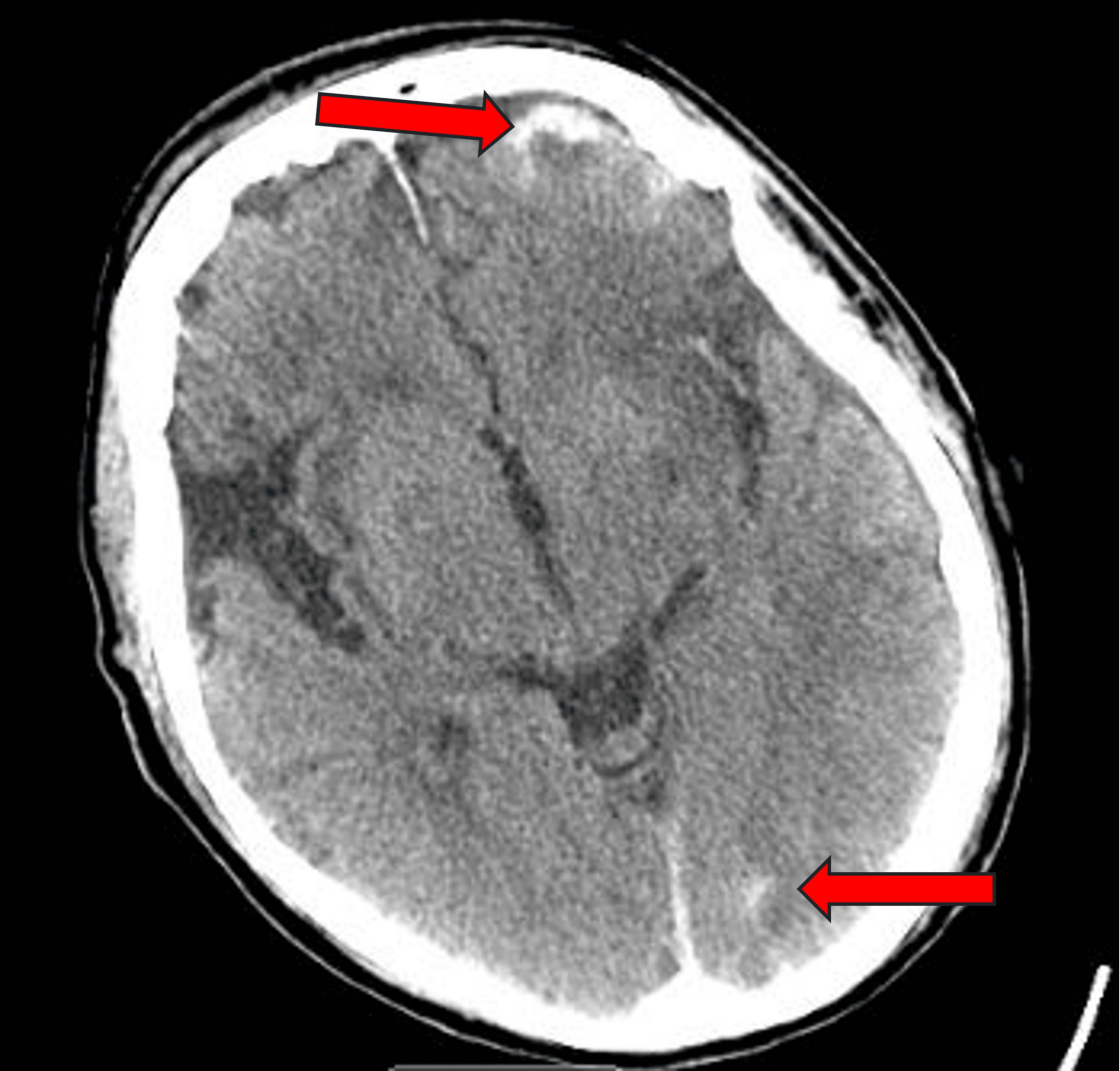
Patient 2 head CT without contrast Subarachnoid hemorrhage to the left frontal lobe, left parietal lobe, and subdural hemorrhage to the left frontal region (top arrow), and a left occipital lobe hemorrhage (bottom arrow).

**Figure 2:**
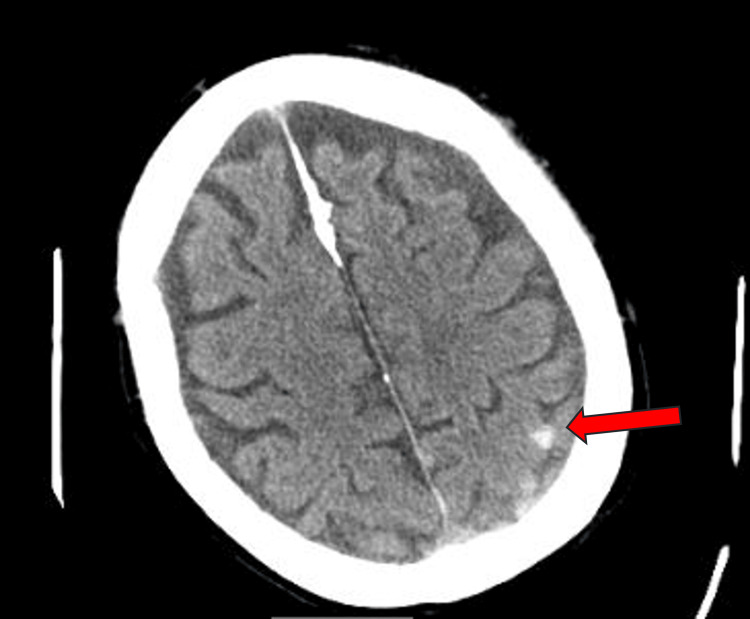
Patient 2 head CT without contrast occipital lobe hemorrhage

Patient 2 is an 89-year-old male with a past medical history of macular degeneration, hypertension, and atrial fibrillation controlled with warfarin who presented to the DRMC emergency department after falling down a casino escalator. Per the patient’s wife, he was consuming alcohol (EtOH level 42mg/dL) before falling down the escalator and losing consciousness and bladder control. He had a laceration of the right eyebrow and left forehead which were both repaired without complications. CT of the head without contrast showed acute subarachnoid hemorrhages of the left frontal, left parietal, and left occipital lobes, as well as left frontal subdural hemorrhage without skull fracture (Figure 1, Figure 2). Neurosurgery and orthopedics were consulted, and factor eight inhibitor bypassing activity (FEIBA) and vitamin K were initiated to reverse the anticoagulant per hospital protocol. He was admitted to the hospital and two days later had sudden onset right-sided weakness of the face, upper, and lower extremities. Repeat CT of the head did not show any acute changes, however CT angiography (CTA) of the head and neck showed a left M1 middle cerebral artery occlusion. No embolectomy was performed, and the patient’s mentation deteriorated, leading to the patient’s family placing him on hospice. He died after spending six days in the hospital, two of which were in the intensive care unit.

Patient 3 is a 74-year-old female with past medical history of hypertension and cerebrovascular accident controlled with clopidogrel who presented to the DRMC emergency department after falling down the airport escalator. She stated she was in normal health and fell while trying to catch her suitcase as it fell down the escalator. She subsequently sustained head trauma and lost consciousness, per emergency medical services. While being evaluated by nursing staff in the ED, she complained of dizziness and lost consciousness again, however, she did not require any intervention. A scalp laceration was repaired with staples, and imaging showed no fractures or acute hemorrhages. She was admitted to the telemetry floor for monitoring, and did not experience any more syncopal episodes. She was discharged home after two days in the hospital with no days spent in the intensive care unit.

Patient 4 is a 77-year-old female with past medical history of chronic obstructive pulmonary disease (COPD) who presented to the DRMC ED after falling down approximately 30 feet on an escalator. She reports falling after someone else had tripped and fallen into her at the casino. She was intoxicated upon admission (EtOH 187mg/dL) and had sustained multiple superficial lacerations to the face and upper extremities, which were repaired by emergency department physicians. Emergency medical services also stated the patient had lost consciousness upon their arrival, and was confused throughout transport. Upon imaging, it was discovered that she had sustained a left apical pneumothorax, so she was admitted for further observation. Two days into her hospital stay, with no days spent in the intensive care unit, the patient left against medical advice.

Patient 5 is a 76-year-old female with a past medical history of hypertension, hyperlipidemia, and coronary artery disease with prior myocardial infarction currently on clopidogrel who presented to the DRMC ED after slipping and falling backward down an escalator. She did not lose consciousness, but did sustain multiple lacerations to the scalp, lower back, and extremities, none of which needed staples or suturing. Imaging showed no traumatic fractures or acute hemorrhages. Trauma team was consulted for evaluation, and the patient was deemed medically safe for discharge from the ED.

Patient 6 is a 70-year-old female with a past medical history of hypertension and diabetes mellitus who presented to the DRMC ED after falling approximately 10 feet down an escalator at the local casino. She denies loss of consciousness, however she did sustain a nasal bone fracture and globe rupture of the right eye, causing her decreased vision. Ophthalmology was consulted and repaired the patient’s eye. She was discharged from the hospital the next day after no days spent in the intensive care unit.

Patient 7 is an 80-year-old female with a history of hypertension, diabetes mellitus, hypothyroidism, and gastroesophageal reflux disease (GERD) who presented to DRMC ED after falling down an escalator. She denies any loss of consciousness, only complaining of right-sided head and neck pain. Imaging was negative for any traumatic fractures or acute hemorrhaging. She was reevaluated approximately four hours after arrival to the ED and was found to be comfortable with no complaints. Patient was sent home from DRMC ED with previous level of function.

**Figure 3:**
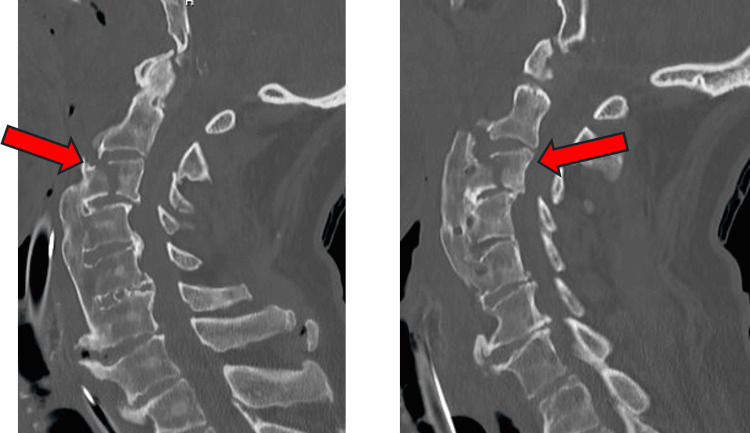
Patient 8 neck CT without contrast Arrows indicating C3 vertebral fracture

Patient 8 is an 84-year-old male with no known past medical history who presented to JFK ED after falling down a casino escalator. Emergency medical services state patient was found unconscious receiving CPR by bystanders. EMS found the patient tachycardic with a pulse, Glasgow Coma Scale (GCS) 3, and agonal respirations. The patient was transferred to DRMC ED by air, where upon arrival rapid sequence intubation (RSI) and a left femoral central venous line was placed. Patient also suffered a left eyebrow laceration and left hemotympanum, leading to suspicion of an open depressed skull fracture. Imaging at DRMC revealed an acute C3 vertebral fracture (Figure 3), fractured left ribs 1 through 7, fractured right ribs 2 through 5, bilateral pleural effusions, and a right ventricular thrombus. Neurosurgery was consulted and it was determined the patient was too ill to undergo surgery, so the family agreed to comfort care measures given the extent of his injuries. He was compassionately extubated four days after entering the intensive care unit and subsequently died from his injuries.

Table 1 shows the patient demographics, anticoagulation status upon admission, and loss of consciousness at emergency medical service’s arrival.

**Table 1:** Patient Demographics M: male; F: female; LOC: loss of consciousness

Patient	Age	Sex	Past Medical History	Anticoagulation	LOC
1	96	F	No known history	No	No
2	89	M	Hypertension, atrial fibrillation	Yes (Warfarin)	Yes
3	74	F	Hypertension, Cerebral Vascular Accident	Yes (Clopidogrel)	Yes
4	77	F	Chronic obstructive pulmonary disease (COPD)	No	Yes
5	76	F	Hypertension, Coronary Artery Disease w/ history of Myocardial Infarction	Yes (Clopidogrel)	No
6	70	F	Hypertension, Diabetes Mellitus	No	No
7	80	F	Hypertension, Diabetes Mellitus, Hypothyroidism	No	No
8	84	M	No known history	No	Yes

Table 2 presents patients' hospital length of stay, days spent in the ICU, injury patterns, and disposition upon discharge.

**Table 2:** Hospital Course, Injury Patterns, Disposition upon Discharge LOS: length of stay; ICU: intensive care unit; R: right; L: left; Fx: fracture; PLOF: previous level of function

Patient	LOS	ICU Days	Injuries	Disposition
1	7	3	Laceration R Upper Extremity, Laceration R Lower Extremity, Laceration L Lower Extremity	PLOF
2	6	2	Laceration R Eyebrow, Laceration L Forehead, Acute Subarachnoid Hemorrhage, Acute Subdural Hemorrhage	Deceased
3	2	0	Laceration Scalp, Concussion	PLOF
4	2	0	L Pneumothorax, Laceration R Hand, Laceration R Upper Extremity	PLOF
5	0	0	Laceration Scalp, Laceration L Upper Extremity, Laceration R Upper Extremity, Laceration L Lower Extremity, Laceration R Lower Extremity, Laceration Lower Back/Pelvis	PLOF
6	2	0	Nasal Fracture, Globe Rupture R Eye, Laceration Face	PLOF
7	1	0	No Trauma Diagnosis	PLOF
8	4	4	Cardiac Arrest, Respiratory Failure, C3 Unstable Fx, T4 Fx, L1 Fx, L Ribs 1-7 Fx, R Ribs 2-5 Fx, b/l Pleural Effusion, Laceration L Eyebrow, L Hemotympanum	Deceased

## Discussion

Falls are the leading cause of unintentional injury in individuals over the age of 65, with escalator-related falls presenting a unique array of injury patterns [1]. The data presented in this retrospective study supplied by Desert Regional Medical Center highlights the significant morbidity associated with falls from escalators among people 65 and older. The average age of patients in this study was 80.75 years, with a median age of 78.5 years, reflecting the vulnerability of this demographic. Of note, 75% (6) of our cases were female while only 25% (2) were male. This finding aligns with existing literature demonstrating that elderly women are at a higher risk of falling compared to males [11].

The outcomes observed in this study shed light on the potential severity of injuries sustained from escalator falls. Two patients died from injuries related to their fall, both of whom were males. All female patients were released from the hospital with expectations of returning to their previous level of function. Interestingly, these statistics are also seen at other Level 1 trauma centers, with one study demonstrating that females older than 65 years fall from height 1.2 times more frequently than males, however males consistently had higher mortality rates than females [11]. Three patients were taking anticoagulant medications prior to their falls - warfarin and clopidogrel. This poses further complications to care, as it can exacerbate bleeding and increase the risk of complications.

Alcohol use may also be a contributing factor to falls from escalators. Two patients presented with elevated blood ethanol levels, both of which experienced their falls in the setting of a casino. Other studies have demonstrated positive correlations between alcohol intoxication and fall risk, further increasing the potential for injury when using escalators [5]. In total, four patients fell from escalators located in casinos, while only one fell from an escalator at an airport. The other three patient locations were unspecified. This potential association highlights the need for targeted safety measures in settings where alcohol is consumed, particularly for older adults who may already have an increased risk of falls.

The average length of stay between these eight patients was three days, with the longest length of stay being seven days. Two patients did not require admission to the hospital based on their injuries, patient 5 and patient 7. Both patients presented without loss of consciousness or lacerations requiring suture or staples for closure. The average length of stay in the intensive care unit was 1.125 days, with the longest length of stay being four days. Among these patients, there were no outliers regarding the length of their hospital stay based on the injuries sustained.

Injury patterns sustained in these patients ranged widely, from simple lacerations that did not require medical attention by emergency physicians to fractures and subdural hemorrhages. The most common injury seen was laceration to the face or scalp, presenting in six of the eight patients. Most patients who presented with at least one facial or scalp lacerations also presented with more than one laceration to that region. The next most common injuries seen were lacerations to the upper and lower extremities, presenting in two patients. Other injuries seen in this patient population include concussion, pneumothorax, fractures to the nasal bones, ribs, and vertebrae, and acute subdural and subarachnoid hemorrhages.

Morbidity is a potential threat to all patients who fall from a height. Our study found two patients, both male, who died of their injuries after falling from an escalator. Patient 2 presented with acute subarachnoid and subdural hemorrhaging and subsequently suffered from a middle cerebral artery stroke. It was found that he had extensive plaque occluding both internal carotid arteries. Of note, this patient was on warfarin at presentation to the emergency department, which was reversed with vitamin K and FEIBA per neurosurgery. It cannot be determined if the cause of death in this patient was a direct result of falling, or from the anticoagulant reversal therapy provided once the patient was hospitalized. In patient 8, who also died from their injuries, it is unknown whether their fall from an escalator was the inciting event causing a cardiac arrest and respiratory failure, or if it was the cause of their fall. Both these patients shed light on the potential catastrophic injuries that can be sustained from escalator-related falls, and the importance of implementing proper safety measures.

Other studies have noted the injury patterns escalators can cause in children. Although studies have shown that people aged 65 and older are the most likely to experience injuries related to escalators, children 15 and younger experience significant traumas from escalators [12]. The most common injuries seen in this age group are typically caused by entrapment of the foot at the end of the escalator. Outcomes included crush injuries to the toes leading to amputation, severe degloving of the foot, open fracture-dislocations, and deep lacerations resulting in severed tendons of the foot [13]. These injuries are vastly different from the injuries sustained by the older population of the Coachella Valley. A potential cause of our hospital not seeing any pediatric escalator-related injuries could be due to the setting. Most of our patients presented from the casinos, a setting that does not allow patrons under 18-21 years.

Other literature discussing escalator-related injuries also reports people aged 65 and older as more vulnerable to experiencing severe injuries, attributing this to previous health conditions correlating to the severity of injury sustained [14]. They found that falls were the main hazard associated with moderate (90%) and minor (91.5%) injuries sustained from escalators. They also found that female passengers were more likely to be injured when falling from an escalator, with their population consisting of 65.9% females and 34.1% males across all ages. Furthermore, the injury patterns sustained across this population occurred mostly to the head and neck (42%) and extremities (42%) [14]. This correlates with what we have seen at our Level 1 trauma center, where 75% of injuries occurred to females and injuries to the head, neck, and extremities were the most common upon presentation.

Although our institution did not obtain details related to the event that incited each individual’s fall, some studies have obtained this data. Through disbursing a 10-question questionnaire to patients who had presented to the hospital after a fall down stairs or escalators, they found that 16% blamed rain, 16% blamed high heels or sandals, and 25% blamed poor visibility [15]. They further discussed patients were more likely to use the handrail, move up and down the escalators slower, and were more likely to look down at their feet while descending. These findings, along with proper education and signage surrounding escalators, could help mitigate future risks in this vulnerable population.

Limitations to this study include incomplete data collection and a small sample size, which may hinder the ability to draw definitive conclusions regarding injury patterns and outcomes. Additionally, lower acuity patients may have been transported to other hospital systems in the Coachella Valley, leading to an underrepresentation of escalator-related injuries in this study. Further research of this subject should aim to include larger sample sizes and multi-center data to better understand the epidemiology of escalator-related traumas and develop effective prevention strategies. 

Here we present eight cases of trauma patients injured after falling while on an escalator. As an uncommon injury mechanism this case series highlights the potential for significant injuries and possibly death from escalator-related injuries.

## Conclusions

Escalator-related injuries pose a significant risk to individuals aged 65 and older. The potential for serious injury, including fractures, hemorrhages, and death, highlights the importance of implementing safety measures in environments where escalators are prevalent. Providing alternative transportation methods, such as elevators, along with proper education and warning signs of the risks associated with escalator use in people of high fall risk, could help mitigate injuries in this vulnerable population. Continued research is essential to further understand the injury patterns associated with falls from escalators, as well as identify public health initiatives aimed at reducing the incidence of such injuries. 
